# Selenium Yeast Attenuated Lipopolysaccharide-Induced Inflammation in Porcine Mammary Epithelial Cells by Modulating MAPK and NF-κB Signaling Pathways

**DOI:** 10.3390/antiox14030334

**Published:** 2025-03-12

**Authors:** Zhenting He, Senlin Su, Bing Zhang, Dongpang Chen, Siyu Yuan, Wutai Guan, Shihai Zhang

**Affiliations:** 1 Guangdong Province Key Laboratory of Animal Nutrition Control, College of Animal Science, South China Agricultural University, Guangzhou 510642, China; hezhenting@stu.scau.edu.cn (Z.H.); susenlin@stu.scau.edu.cn (S.S.); 1760662690@stu.scau.edu.cn (B.Z.); dongpangchen@stu.scau.edu.cn (D.C.); yuansiyu@stu.scau.edu.cn (S.Y.); wtguan@scau.edu.cn (W.G.); 2 National Engineering Research Center for Breeding Swine Industry, South China Agricultural University, Guangzhou 510642, China; 3 Guangdong Laboratory for Lingnan Modern Agriculture, South China Agricultural University, Guangzhou 510642, China

**Keywords:** porcine mammary epithelial cells, anti-inflammation, selenium yeast, milk synthesis, lipopolysaccharide

## Abstract

Mastitis, a prevalent inflammatory disease in mammals, disrupts mammary gland function, compromises milk quality, and can contribute to increased offspring morbidity and mortality. Maintaining the health of porcine mammary epithelial cells (PMECs), the primary cell type in the mammary gland, is crucial for minimizing the adverse effects of this disease. Selenium yeast (SeY), an organic selenium compound known for its antioxidant and immune-enhancing properties, has yet to be fully understood in its role in modulating inflammation in mammary gland. In this study, lipopolysaccharide (LPS) (50 µg/mL, 24 h) significantly upregulated the expression of pro-inflammatory cytokines, including tumor necrosis factor-alpha (TNF-α), interleukin-6 (IL-6), interleukin-8 (IL-8), and interleukin-1β (IL-1β) (*p* < 0.05). Pretreatment with 1 µM SeY significantly attenuated the LPS-induced inflammatory response by reducing the levels of TNF-α, IL-6, IL-8, and IL-1β (*p* < 0.05). Additionally, SeY enhanced cellular antioxidant defenses by increasing total antioxidant capacity (T-AOC), superoxide dismutase (SOD) activity, glutathione (GSH) levels, and glutathione peroxidase (GSH-Px) activity, while concurrently decreasing malondialdehyde (MDA) accumulation (*p* < 0.05). SeY also restored both intracellular and extracellular triglyceride levels and rescued lipid droplet formation, which were disrupted by LPS treatment. Furthermore, SeY upregulated key regulators involved in milk synthesis (*p* < 0.05). These findings suggest that SeY effectively mitigates LPS-induced inflammation and oxidative stress while preserving critical pathways for milk fat and protein synthesis in PMECs.

## 1. Introduction

Mastitis is a prevalent and significant disease in animal production, often leading to reduced milk production or altered milk composition. This condition can severely affect the growth of offspring, sometimes resulting in piglet mortality, and causing substantial economic losses to the breeding industry [1,2,3,4,5,6,7,8]. Mammary epithelial cells, the primary cellular component of the mammary gland, play a crucial role in the inflammatory response, serving as both targets and mediators of infection-induced inflammation in mastitis [9,10,11]. Lipopolysaccharide (LPS), a key component of Gram-negative bacterial cell walls, triggers intracellular signaling cascades by binding to Toll-like receptor 4 (TLR4) on the cell surface, leading to the activation of pro-inflammatory pathways [12,13,14]. This activation initiates both MyD88-dependent and MyD88-independent pathways, culminating in the activation of nuclear factor-kappa B (NF-κB) [15]. Once activated, NF-*κ*B promotes the transcription and release of pro-inflammatory factors, including cytokines and chemokines [16], which orchestrate immune responses and drive inflammation in mammary epithelial cells [7,17,18]. Given its pivotal role in inflammatory cascade, the LPS-induced signaling pathway represents a critical target for identifying potential therapeutic agents to mitigate mammary gland inflammation.

Selenium, an essential trace element, plays a crucial role in various biological processes, particularly as a component of antioxidant enzymes that neutralize reactive oxygen species (ROS) and enhance immune function [19,20,21]. Previous studies have demonstrated that selenium supplementation improves the reproductive performance and health status of sows and their offspring [22,23,24]. Increased maternal selenium intake has been shown to enhance piglet survival, colostrum and milk quality, maternal antioxidant status, and immunoglobulin transfer [25]. Selenium also supports piglet growth during early lactation [26,27]. In animal feed, selenium is available in both inorganic and organic forms [28]. Organic selenium has been shown to provide several advantages over inorganic forms, including higher absorption rates [29], enhanced antioxidant capacity [30], lower toxicity [31,32], and improved overall animal health and performance [33,34,35,36,37]. Among various organic selenium sources, selenium yeast (SeY) offers distinct advantages, including superior bioavailability and enhanced antioxidant capacity, whereas selenium methionine (Sel-Met), despite its good absorption, exhibits relatively lower stability and bio-conversion efficiency. Previous studies in our lab systematically compared the antioxidant efficacy of SeY and Sel-Met in porcine mammary epithelial cells (PMECs), demonstrating that both enhance antioxidant capacity through activation of the p38/JNK signaling pathway, underscoring selenium’s critical role in mitigating oxidative stress in the mammary glands of sows and their offspring.

Despite the well-documented benefits of SeY, most studies have primarily focused on its general role in enhancing overall animal health. However, its specific protective effects against LPS-induced inflammation and oxidative stress in PMECs remain largely unexplored. This study provides novel insights into the potential of SeY in mitigating LPS-induced inflammatory responses and oxidative stress in PMECs. Additionally, we elucidate the underlying mechanisms by which SeY regulates key signaling pathways, including NF-κB and mitogen-activated protein kinase (MAPK) pathways, and its impact on milk fat and protein synthesis pathways. These findings offer new perspectives on the therapeutic potential of SeY for mastitis prevention and management, emphasizing its role in improving lactation and overall mammary gland health in livestock.

## 2. Materials and Methods

### 2.1. Preparation of SeY

SeY from Sel-Plex™ 2000 (Alltech Inc., Lexington, KY, USA), containing 2000 mg/kg of selenium, was used in this study. To simulate the gastrointestinal digestion of SeY, SeY was subjected to pretreatment with digestive enzymes in vitro. A protease solution was prepared by dissolving 2 mg of protease XIV (Sigma-Aldrich, Saint Louis, MO, USA) in 0.5 mL of 10 mM Tris-HCl buffer (Sigma-Aldrich, Saint Louis, MO, USA). To this solution, 40 mg of SeY was added and thoroughly mixed. The samples were disrupted using ultrasound (25 s at 80% amplitude) on ice, followed by cleaning with ultrapure water (Sangon Biotech, Shanghai, China). The ultrasonic power was set to 30 W, and the disruption lasted for 15 min. The sample was then centrifuged at 14,000 rpm for 3 min, and the supernatant was discarded. The pellet was washed, resuspended in ultrapure water (Sangon Biotech, Shanghai, China), and centrifuged again under the same conditions to obtain the final supernatant. The selenium concentration in the sample was determined by inductively coupled plasma mass spectrometry (ICP-MS).

### 2.2. Cell Culture

PMECs were isolated from the mammary gland of a 9-month-old Large White sow [38]. The sow was selected based on her optimal health status and body condition. PMECs isolated from a lactating sow were cryopreserved in liquid nitrogen at −196 °C. For culture, thawed PMECs (1 mL, 37 °C) were maintained in DMEM/F12 medium supplemented with 10% FBS, 1% antibiotic-antimycotic, 10 ng/mL IGF-1, 10 ng/mL EGF, 5 µg/mL ITS, and 5 µg/mL hydrocortisone at 37 °C with 5% CO_2_. The cells were subsequently transferred to DIP medium supplemented with 1 μM dexamethasone, 5 μg/mL insulin, and 5 μg/mL prolactin to induce differentiation and stimulate milk production.

### 2.3. Cell Viability Assay

Cell viability was assessed using the CCK-8 assay (Nanjing Jiancheng Bioengineering Institute, Nanjing, China). PMECs were seeded in 96-well plates at 1 × 10^5^ cells/mL (100 µL medium/well) and incubated at 37 °C with 5% CO_2_ for 24 h. At 70–80% confluence, cells were treated as specified. Post-treatment, 10 µL of CCK-8 solution was added to each well, incubated at 37 °C for 1–3 h, and the absorbance was measured at 450 nm using a microplate reader.

### 2.4. Real-Time PCR

Total RNA was extracted from the cell samples using the EZ-press RNA purification kit (EZ-Bio, Shanghai, China). cDNA synthesis was performed using the RNA reverse transcription kit (EZ-Bio, Shanghai, China). The cDNA was mixed with Color SYBR Green qPCR Mix, target gene primers, and double-distilled water to prepare a 20 µL qPCR reaction system. The thermal cycling conditions were as follows: 95 °C for 1 min, followed by 40 cycles of 95 °C for 15 s, 59 °C for 15 s, and 72 °C for 40 s. Relative gene expression was calculated using the 2^−ΔΔCt^ method, with β-actin as the internal control. Primer sequences for real-time PCR are listed in Table 1.

### 2.5. Measurement of Inflammatory Factor Levels

The levels of tumor necrosis factor-alpha (TNF-α), interleukin-6 (IL-6), interleukin-8 (IL-8), and interleukin-1β (IL-1β) were quantified using pig-specific ELISA kits (mlbio, Shanghai, China). PMECs were seeded in 12-well plates at 2.5 × 10^4^ cells/mL (1 mL/well) and cultured at 37 °C with 5% CO_2_. After 48 h, cells were pretreated with 1 µM SeY for 24 h, followed by 50 µg/mL LPS stimulation for 24 h. The cells were washed with PBS, lysed in 140 µL RIPA buffer (Beyotime, Shanghai, China), and centrifuged (10,000 rpm, 10 min). Supernatants were collected for analysis.

### 2.6. Antioxidant Enzymes Assay

Antioxidant capacity was assessed using a commercial kit (Nanjing Jiancheng Bioengineering Institute, Nanjing, China). PMECs were seeded in 12-well plates at 2.5 × 10^4^ cells/mL (1 mL/well) and cultured at 37 °C with 5% CO_2_. After 48 h, cells were pretreated with 1 µM SeY for 24 h, followed by 50 µg/mL LPS stimulation for 24 h. The LPS concentration (50 μg/mL) and exposure time (24 h) used in this study were determined based on prior experiments conducted in our lab [39]. The cells were washed with PBS, lysed in 140 µL RIPA buffer (Beyotime, China), and centrifuged (12,000 rpm, 5 min). Supernatants were analyzed for total antioxidant capacity (T-AOC), superoxide dismutase (SOD), malondialdehyde (MDA), glutathione (GSH) and glutathione peroxidase (GSH-Px) levels.

### 2.7. Western Blot Analysis

PMECs were seeded in 12-well plates at 2.5 × 10^4^ cells/mL (1 mL/well) and cultured at 37 °C with 5% CO_2_ for 48 h. Cells were treated with 1 μM SeY for 24 h, followed by 50 μg/mL LPS for 24 h. After treatment, the cells were washed with PBS, and proteins were extracted using RIPA buffer (Beyotime, Shanghai, China). Protein concentrations were measured with a BCA Protein Assay Kit (Beyotime, Shanghai, China). Equal protein (10–20 µg) was separated by SDS-PAGE (Invitrogen, Carlsbad, CA, USA) and transferred to nitrocellulose membranes (Millipore, Bedford, MA, USA), followed by incubation with primary antibodies (Table 2).

### 2.8. Statistical Analysis

Data were analyzed using IBM SPSS 26.0 with one-way ANOVA and LSD post-hoc tests for group comparisons. GraphPad Prism 8.0 was used for additional analyses and visualizations. Significance was set at *p* < 0.05, with *p* < 0.01 indicating high significance.

## 3. Results

### 3.1. Viability of PMECs

**Figure 1:**
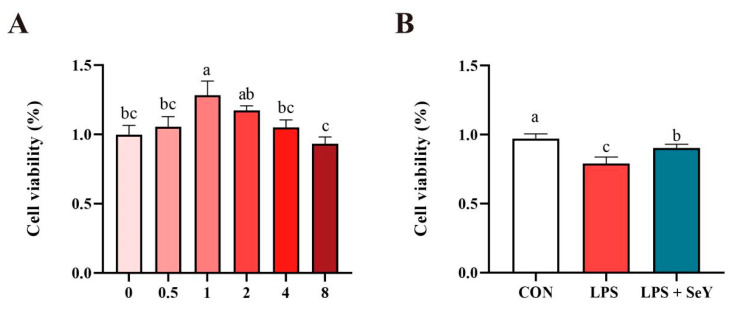
Effect of selenium yeast (SeY) on cell viability of porcine mammary epithelial cells (PMECs). (**A**) Viability of cells treated with varying concentrations of SeY (0, 0.5, 1, 2, 4, and 8 μM) for 24 h. (**B**) Cell viability was assessed using the CCK-8 assay. Bars labeled with different letters (a, b, and c) indicate significant differences between groups (*p* < 0.05). Data are presented as means ± SEM (*n* = 6). Experimental groups: CON (control, untreated cells), LPS (cells treated with 50 μg/mL LPS) and LPS + SeY (cells pretreated with 1 μM SeY followed by 50 μg/mL LPS).

To assess SeY’s effect on PMECs viability, cells were exposed to 0, 0.5, 1, 2, 4, or 8 μM SeY for 24 h, and their viability was assessed using the CCK-8 assay. As illustrated in Figure 1A, viability increased dose-dependently, peaking at 1 μM, which was selected as the optimal pretreatment concentration. Figure 1B shows that LPS significantly reduced viability compared to the controls, but pretreatment with 1 μM SeY partially restored viability (*p* < 0.05).

### 3.2. Inflammatory Factors

**Figure 2:**
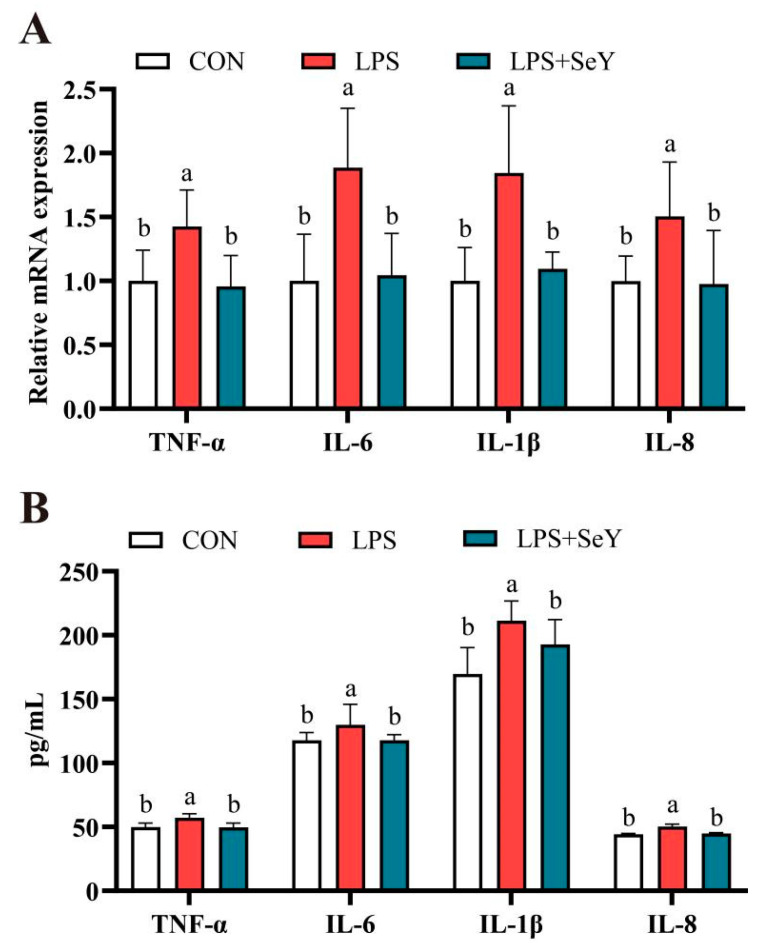
Effect of SeY on the mRNA expression and protein levels of inflammatory cytokines in LPS-induced PMECs. (**A**) mRNA expression levels of inflammatory cytokines. (**B**) Concentrations of inflammatory cytokines. Data are presented as means ± SEM (*n* = 6). Bars labeled with different letters (a, b) indicate significant differences between groups (*p* < 0.05). Experimental groups: CON (control, untreated cells), LPS (cells treated with 50 μg/mL LPS), and LPS + SeY (cells pretreated with 1 μM SeY followed by 50 μg/mL LPS).

Figure 2 shows that LPS significantly upregulated the mRNA expression levels of *TNF-α*, *IL-6*, *IL-1β*, and *IL-8* compared to the controls (*p* < 0.05). Consistently, ELISA results confirmed that LPS also increased the levels of these inflammatory cytokines (*p* < 0.05). Pretreatment with SeY significantly reduced both mRNA expression and concentrations of these inflammatory cytokines, bringing levels close to the controls (*p* < 0.05).

### 3.3. Antioxidant Levels

**Figure 3:**
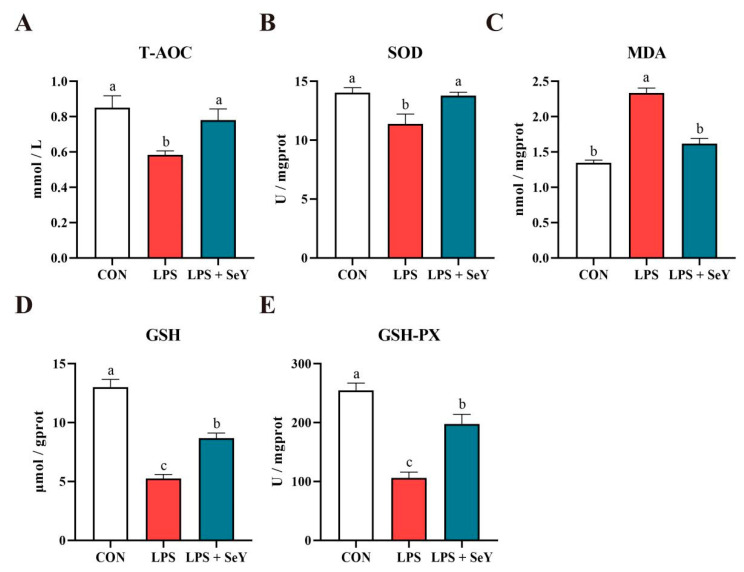
Effect of SeY on the antioxidant capacity of LPS-induced PMECs. (**A**) Levels of total antioxidant capacity (T-AOC), (**B**) superoxide dismutase (SOD), (**C**) malondialdehyde (MDA), (**D**) glutathione (GSH), and (**E**) glutathione peroxidase (GSH-Px) were measured using antioxidant detection kits. Data are presented as means ± SEM (*n* = 6). Bars labeled with different letters (a, b, and c) indicate significant differences between groups (*p* < 0.05). Experimental groups: CON (control, untreated cells), LPS (cells treated with 50 μg/mL LPS), and LPS + SeY (cells pretreated with 1 μM SeY followed by 50 μg/mL LPS).

Figure 3 shows that LPS treatment significantly decreased T-AOC, SOD, GSH, and GSH-Px, while markedly increasing MDA levels (*p* < 0.05). Pretreatment with SeY effectively restored T-AOC, SOD, and MDA levels to near-control values (*p* > 0.05). However, GSH and GSH-Px levels remained intermediate, showing significant differences from both the LPS-only and control groups (*p* < 0.05), suggesting a partial but not complete recovery of antioxidant capacity.

### 3.4. NF-κB and MAPK Signaling Pathways

**Figure 4:**
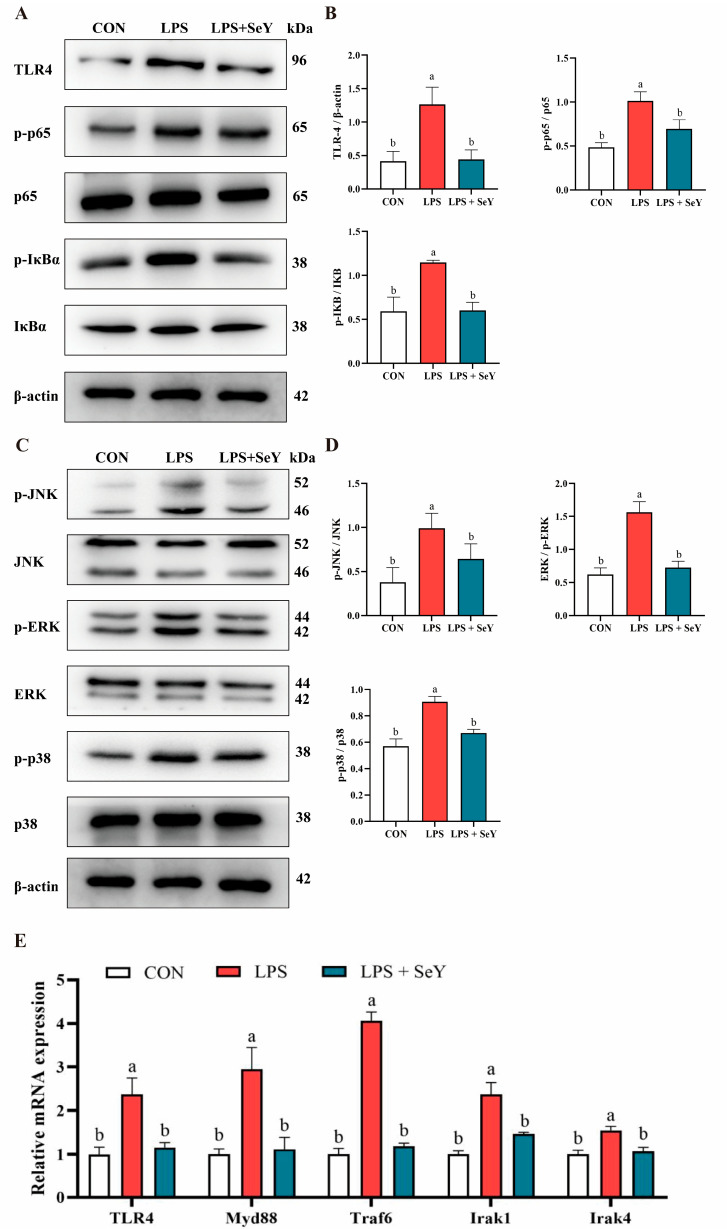
Phosphorylation levels of NF-κB and MAPK signaling pathway proteins in PMECs. (**A**,**B**) Phosphorylation levels of key NF-κB signaling pathway proteins in PMECs. (**C**,**D**) Phosphorylation levels of key MAPK signaling pathway proteins in PMECs. (**E**) mRNA expression levels of NF-κB pathway-related genes in PMECs. Data are presented as means ± SEM (*n* = 3). Different superscript letters (a, b) indicate significant differences between groups (*p* < 0.05). Experimental groups: CON (control, untreated cells), LPS (cells treated with 50 μg/mL LPS), and LPS + SeY (cells pretreated with 1 μM SeY followed by 50 μg/mL LPS).

LPS treatment markedly enhanced the phosphorylation of key NF-κB (IκBα and p65) and MAPK (JNK, ERK, and p38) signaling proteins compared to the control group (*p* < 0.05), indicating robust activation of these inflammatory pathways (Figure 4A–D). SeY pretreatment effectively suppressed this phosphorylation, restoring levels close to those of the controls (*p* < 0.05), suggesting its inhibitory effect on LPS-induced NF-κB and MAPK activation (Figure 4A–D). Similarly, SeY pretreatment significantly attenuated the LPS-induced upregulation of *Myd88*, *Irak4*, *Irak1*, and *Traf6* mRNA expression (*p* < 0.05), further demonstrating its regulatory role in suppressing TLR4-mediated inflammatory signaling (Figure 4E).

### 3.5. Intracellular and Extracellular Triglyceride Levels in PMECs

**Figure 5:**
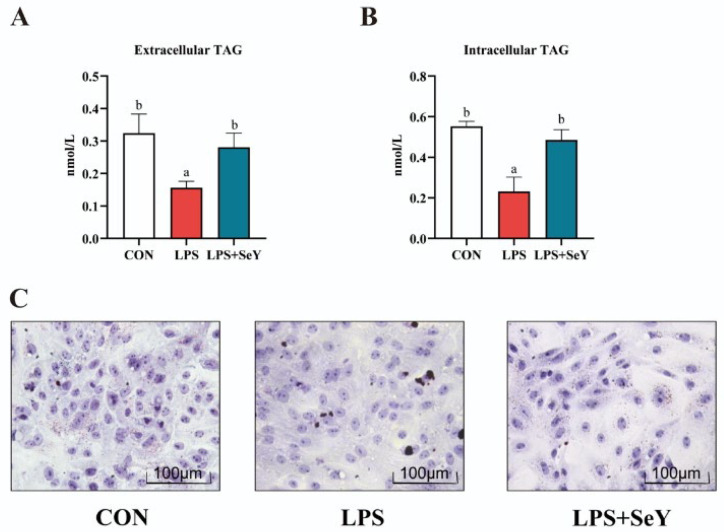
Effect of SeY on intracellular and extracellular triglyceride levels and lipid droplet formation in LPS-induced PMECs. (**A**) Extracellular triglyceride content in PMECs. (**B**) Intracellular triglyceride (TAG) content in PMECs. (**C**) Oil Red O staining showing lipid droplet formation in PMECs. Data are presented as means ± SEM (*n* = 3). Bars labeled with different letters (a, b) indicate significant differences between groups (*p* < 0.05). Experimental groups: CON (control, untreated cells), LPS (cells treated with 50 μg/mL LPS), and LPS + SeY (cells pretreated with 1 μM SeY followed by 50 μg/mL LPS).

LPS treatment significantly decreased intracellular (Figure 5A) and extracellular triglyceride levels (Figure 5B) compared to the controls (*p* < 0.05). However, in the LPS + SeY group, triglyceride levels were restored to near-control levels (*p* < 0.05). Oil Red O staining (Figure 5C) further confirmed that LPS exposure suppressed lipid droplet formation, while SeY pretreatment partially rescued this effect. These findings suggest that LPS disrupts triglyceride synthesis and lipid storage in PMECs, whereas SeY pretreatment counteracts these impairments, thereby promoting lipid accumulation.

### 3.6. mRNA and Protein Expression Related to Milk Fat and Protein Synthesis

**Figure 6:**
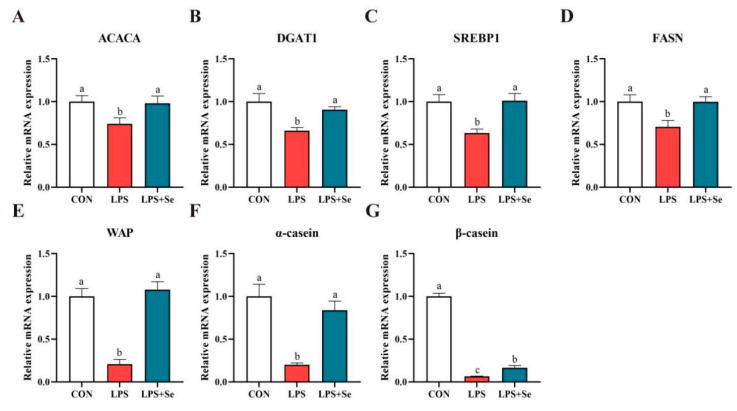
Effect of SeY on the mRNA expression of genes related to milk fat and milk protein synthesis in LPS-induced PMECs. mRNA expression levels of acetyl-CoA carboxylase alpha (ACACA) (**A**), diacylglycerol O-acyltransferase 1 (DGAT1) (**B**), sterol regulatory element-binding protein 1 (SREBP1) (**C**), fatty acid synthase (FASN) (**D**), whey acidic protein (WAP) (**E**), alpha-casein (α-casein) (**F**), and beta-casein (β-casein) (**G**) in PMECs. Data are presented as means ± SEM (*n* = 3). Bars labeled with different letters (a, b, and c) indicate significant differences between groups (*p* < 0.05). Experimental groups: CON (control, untreated cells), LPS (cells treated with 50 μg/mL LPS), and LPS + SeY (cells pretreated with 1 μM SeY followed by 50 μg/mL LPS).

**Figure 7:**
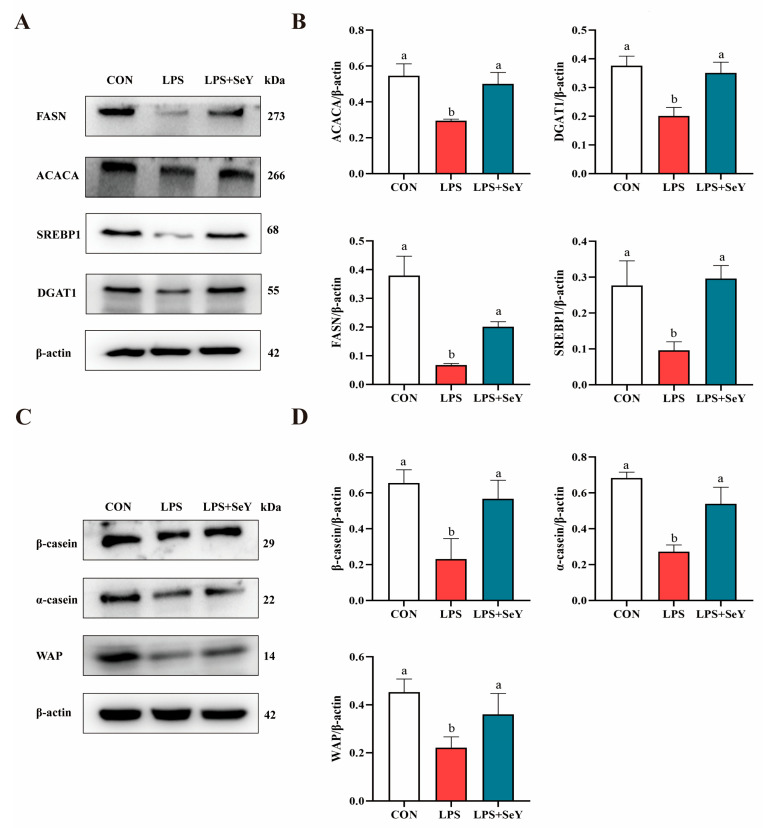
Effect of SeY on the milk fat and milk protein synthesis in LPS-induced PMECs. (**A**,**B**) Protein expression levels of key regulators involved in milk fat in PMECs. (**C**,**D**) Protein expression levels of key regulators involved in milk protein synthesis in PMECs. Data are presented as means ± SEM (*n* = 3). Bars labeled with different letters (a, b) indicate significant differences between groups (*p* < 0.05). Experimental groups: CON (control, untreated cells), LPS (cells treated with 50 μg/mL LPS), and LPS + SeY (cells pretreated with 1 μM SeY followed by 50 μg/mL LPS).

LPS significantly downregulated the mRNA expression of key molecules involved in milk fat and protein synthesis, including *ACACA*, *DGAT1*, *SREBP1*, *FASN*, *WAP*, *α-casein* and *β-casein*, compared to the control group (Figure 6, *p* < 0.05). In the LPS + SeY group, mRNA levels of these molecules were significantly higher than those in the LPS group (*p* < 0.05). Consistently, the protein expression of these molecules was also significantly higher in the SeY-pretreated cells than in the LPS group (Figure 7A,B). These results suggest that SeY effectively counteracts LPS-induced suppression of milk fat and protein synthesis.

### 3.7. Pathways Related to Milk Fat and Protein Synthesis

**Figure 8:**
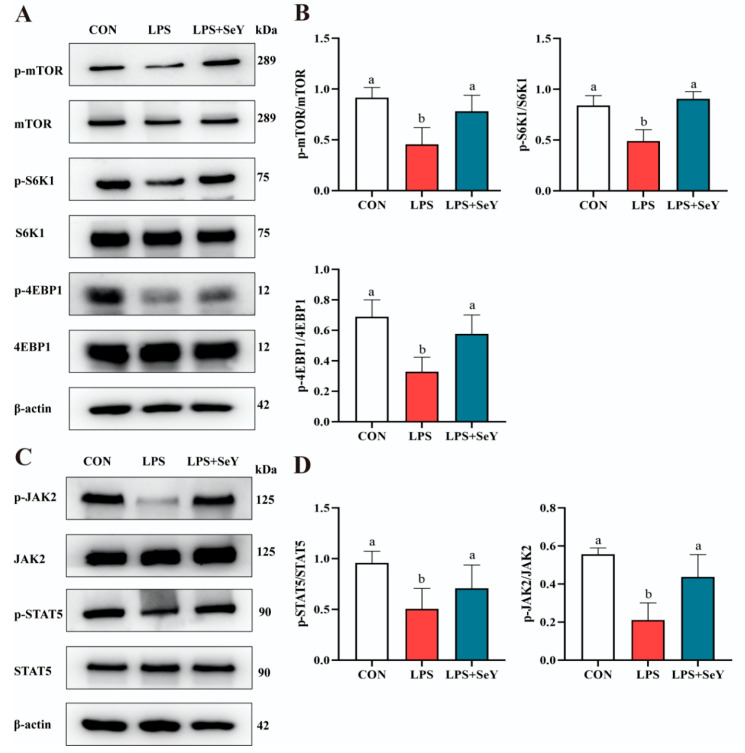
Impact of SeY on milk synthesis signaling pathways in LPS-induced PMECs. (**A**,**B**) Activation of the mechanistic target of rapamycin (mTOR) signaling pathway in PMECs. (**C**,**D**) Activation of the Janus kinase 2-signal transducer and activator of transcription 5 (JAK2-STAT5) signaling pathway in PMECs. The data are the means ± SEM (*n* = 3). Bars labeled with different letters (a, b) indicate significant differences between groups (*p* < 0.05). Experimental groups: CON (control, untreated cells), LPS (cells treated with 50 μg/mL LPS), and LPS + SeY (cells pretreated with 1 μM SeY followed by 50 μg/mL LPS).

To further explore the regulatory mechanisms underlying milk fat and protein synthesis, we examined the mechanistic target of rapamycin (mTOR) and Janus kinase 2-signal transducer and activator of transcription 5 (JAK2-STAT5) pathways. Western blot analysis showed that LPS treatment significantly reduced phosphorylation levels of mTOR, S6K1, 4EBP1, STAT5, and JAK2 compared to the control group (*p* < 0.05) (Figure 8A–D). However, SeY pretreatment significantly restored the phosphorylation of these proteins (*p* < 0.05), bringing them to levels comparable to those of the controls (Figure 8A–D).

## 4. Discussion

Mastitis is a significant concern in dairy cattle, as it directly impacts milk production and quality, leading to substantial economic losses in the dairy industry [40]. Extensive research has focused on anti-inflammatory strategies, such as vitamin E and selenium supplementation, which have demonstrated beneficial effects in modulating immune responses and alleviating oxidative and inflammatory stress in perinatal cows [41]. However, compared to dairy cattle, mastitis in sows has received relatively less research attention, despite its severe consequences, including maternal inflammation, impaired lactation, and increased piglet mortality [42]. This underscores the urgent need for further research into effective preventive and therapeutic strategies for sow mastitis.

Inflammation is a complex and essential defense mechanism triggered by various stimuli, including microbial infection and tissue damage [43,44]. In the mammary gland, excessive inflammatory responses can disrupt normal metabolic functions, compromise milk synthesis, and negatively affect overall lactation performance. Enhancing immune function and anti-inflammatory capacity is therefore critical for maintaining mammary gland health and sustaining optimal milk production. LPS, a key component of Gram-negative bacterial cell walls, is a well-established inflammatory inducer that severely disrupts mammary gland homeostasis. LPS exposure impairs mammary epithelial cell function by triggering oxidative stress, reducing antioxidant capacity, and negatively affecting milk yield and composition [4,11,45,46]. Mechanistically, LPS binds to TLR4 on the cell surface [47], initiating a cascade of inflammatory signaling events [39,48]. In this study, SeY was evaluated for its protective effects on PMECs. A dose-dependent response in cell viability was observed, with the highest viability detected at 1 µM SeY after 24 h. Pretreatment with 1 µM SeY for 24 h, followed by 50 µg/mL LPS exposure for 24 h, significantly improved cell viability compared to the LPS group. These results suggest that SeY mitigates LPS-induced viability reduction, likely through its antioxidant and anti-inflammatory properties, thereby supporting PMEC resilience under inflammatory conditions.

Oxidative stress results arises from an imbalance between reactive oxygen species (ROS) production and the body’s antioxidant defense mechanisms and is closely linked to inflammation [49,50]. Selenium plays a critical role in repairing oxidative stress-induced damage and enhancing cellular antioxidant capacity [51]. Previous studies have shown that SeY regulates selenoprotein expression and enhances antioxidant capacity in PMECs via activation of the p38/JNK signaling pathway, thereby promoting cell viability [32,52]. In this study, SeY pretreatment significantly increased T-AOC, SOD, GSH, and GSH-Px levels while reducing MDA accumulation in LPS-induced inflammatory cells. These results are consistent with those of Yang et al. [53], who reported that hydroxy-selenomethionine (HMSeBA) can help dairy cows overcome heat stress by enhancing antioxidant capacity. By restoring redox balance, SeY not only mitigates oxidative stress-induced damage but also suppresses inflammatory responses.

Earlier research demonstrated that selenium pretreatment suppressed the expression of pro-inflammatory genes, such as *TNF*-*α* and *COX*-*2*, by inhibiting NF-*κ*B p65, I*κ*B*α*, p38, ERK, and JNK phosphorylation in mammary epithelial cells [48]. Similarly, selenium reduced the gene expression of inflammatory cytokines (*TNF*-*α*, *IL*-*6β*, and *IL*-*2*) in Staphylococcus aureus-stimulated bovine mammary epithelial cells by modulating TLR4, NF-*κ*B, and MAPK signaling pathways [54]. In alignment with previous findings, the present study demonstrates that pretreatment with SeY markedly decreased both the mRNA levels and protein expression of pro-inflammatory cytokines in PMECs subjected to LPS-induced inflammatory conditions.

The NF-*κ*B signaling system is critical in cellular responses to stimuli such as stress, pro-inflammatory cytokines, free radicals, and heavy metals. The dysregulation of this pathway is closely associated with inflammatory diseases [55]. Additionally, the MAPK family serves as a critical downstream signaling hub for various growth factor receptors and pattern recognition receptors. These kinases are frequently hyperactivated during inflammation, leading to the amplification of pro-inflammatory signaling cascades [56]. The excessive production of inflammatory factors is a primary contributor to cell and tissue damage during inflammation. Previous studies have shown that Se inhibited the LPS-induced inflammatory response by suppressing NF-κB and MAPK in mammary epithelial cells and bovine endometrial epithelial cells [48,57]. In line with these findings, our study demonstrates that SeY similarly exerts anti-inflammatory effects by targeting the NF-κB and MAPK signaling pathways in PMECs. Specifically, SeY reduced the mRNA expression of key NF-κB pathway genes, including *Myd88*, *Irak1*, *Irak4*, and *Traf6*, and restored phosphorylation levels of NF-κB and MAPK pathway proteins (p65, IκBα, p38, ERK, and JNK) to levels comparable to the control group. These results not only confirm the conserved anti-inflammatory mechanism of selenium across different cell types but also provide novel insights into the specific molecular targets of SeY in mammary epithelial cells.

The health and structural integrity of the mammary gland are essential for female livestock to maintain optimal lactation performance. Lactation ability and milk quality are directly linked to the growth rate and survival of offspring [58]. Research has shown that dietary supplementation with 0.3 mg/kg SeY enhances lactation performance in lactating donkeys and improves milk protein production efficiency [59]. Similarly, our laboratory previously demonstrated that the addition of yeast culture combined with organic selenium increased the capacity for milk fat synthesis in lactating sows [32]. Milk fat and protein synthesis are critical physiological functions of mammary epithelial cells [60]. Inflammation can inhibit milk synthesis by disrupting related signaling pathways [61]. In this study, SeY alleviated inflammation and enhanced the expression of milk fat and protein synthesis-related molecules. The mTOR and JAK2-STAT5 pathways are essential for mammary development and milk synthesis [62]. SeY pretreatment consistently alleviated the inhibitory effects of LPS, significantly restoring the phosphorylation levels of the mTOR and JAK2-STAT5 signaling pathways. These results align with previous studies, which demonstrated that organic selenium supplementation in goats notably increases milk fat and protein levels [63]. Together, these findings suggest that SeY influences milk fat and protein synthesis by modulating key signaling pathways.

## 5. Conclusions

In this study, SeY effectively mitigated LPS-induced inflammation and oxidative stress in PMECs, as evidenced by reduced inflammatory cytokine expression and enhanced antioxidant defense. SeY also preserved milk fat and protein synthesis pathways, suggesting its potential to support lactation performance. However, this in vitro study has limitations, particularly in its applicability to the complex in vivo environment, and further research is needed to confirm these findings in animal models. Future studies should explore the molecular mechanisms underlying SeY’s effects, investigate its long-term impact on mammary gland health, and evaluate its potential in combination with other nutritional interventions to optimize sow productivity and reduce mastitis incidence.

## Figures and Tables

**Table 1:** Primer sequences for the mRNA.

Genes	Accession	Sequence Primers (5′–3′)	Size (bp)
*TNF-α*	NM_214022.1	F-ATGGGCTGTACCTCATCTACTC	141
		R-GGCTCTTGATGGCAGAGAGG	
*IL-6*	NM_214399.1	F-TGGCTACTGCCTTCCCTACC	132
		R-CAGAGATTTTGCCGAGGATG	
*IL-1β*	XM_021081828.1	F-CCGAAGAGGGACATGGAGAA	88
		R-AGTTGGGGTACAGGGCAGAC	
*IL-8*	NM_213867.1	F-AGGACCAGAGCCAGGAAGAGAC	108
		R-CACAGAGAGCTGCAGAAAGCAG	
*TLR4*	NM_001113039.2	F-CCTGGATGATGTTAGCAGCGATGG	124
		R-GACGAAGACTGGGTGAGGAATGAAC	
*Myd88*	NM_001099923.1	F-TTCTGATGGGCACCTGGAGAGAG	141
		R-CGTCTGGTCCATTGCTAGTGAACTC	
*Irak1*	XM_003135490.4	F-GGTGGAAGAGGAGGCTGAGGAG	113
		R-CGACGATCTGGGCAGCAATGG	
*Irak4*	NM_001112693.1	F-CAGCCCTGTTGTTCACGTAGCC	90
		R-TTGATGAGCGACCCGTTTCTATTGG	
*Traf6*	XM_005652801.2	F-CAACAGCCAGAGGAAATCCGAGAC	148
		R-CACGCCACCTGCAAGAGAATACC	
*β-actin*	XM_021086047.1	F-TGCGGGACATCAAGGAGAAG	176
		R-AGTTGAAGGTGGTCTCGTGG	

**Table 2:** Information related to primary and secondary antibodies in the Western blot.

Antibody	Company	Code No.	Dilution
TLR4	Proteintech (Rosemont, IL, USA)	66350-1	1:1000
NF-*κ*B	CST (Hong Kong, China)	4764S	1:1000
p-NF-*κ*B	CST	3033S	1:1000
I*κ*B	Abcam (Cambridge, UK)	ab32518	1:1000
p-I*κ*B	Abcam	ab133462	1:1000
p38	Proteintech	66234-1	1:1000
p-P38	CST	4092S	1:1000
JNK	CST	9252S	1:1000
p-JNK	CST	4668S	1:1000
ERK	CST	9102S	1:1000
p-ERK	CST	9101S	1:1000
*β*-actin	Abcam	ab8226	1:3000
Goat Anti-Rabbit IgG	Zenbio (Durham, NC, USA)	511203	1:5000
Goat Anti-Mous IgG	Zenbio	511103	1:5000

## Data Availability

The original contributions generated for this study are included in the article; further inquiries can be directed to the corresponding author.
